# Direct Observation of Structural Deformation Immunity for Understanding Oxygen Plasma Treatment-Enhanced Resistive Switching in HfO_*x*_-Based Memristive Devices

**DOI:** 10.3390/nano9101355

**Published:** 2019-09-21

**Authors:** Dong Wang, Shaoan Yan, Qilai Chen, Qiming He, Yongguang Xiao, Minghua Tang, Xuejun Zheng

**Affiliations:** 1 School of Mechanical Engineering, Xiangtan University, Xiangtan 411105, China; 2 Key Laboratory of Welding Robot and Application Technology of Hunan Province, School of Mechanical Engineering, Xiangtan University, Xiangtan 411105, China; 3 Key Laboratory of Microelectronics Devices and Integration Technology, Institute of Microelectronics, Chinese Academy of Sciences, Beijing 100029, China; 4 School of Materials Science and Engineering, Xiangtan University, Xiangtan 411105, China

**Keywords:** resistive switching, HfO_*x*_, oxygen plasma treatment, oxygen ions migration, oxygen vacancy

## Abstract

Oxygen ions’ migration is the fundamental resistive switching (RS) mechanism of the binary metal oxides-based memristive devices, and recent studies have found that the RS performance can be enhanced through appropriate oxygen plasma treatment (OPT). However, the lack of experimental evidence observed directly from the microscopic level of materials and applicable understanding of how OPT improves the RS properties will cause significant difficulties in its further application. In this work, we apply scanning probe microscope (SPM)-based techniques to study the OPT-enhanced RS performance in prototypical HfO_*x*_ based memristive devices through in situ morphology and electrical measurements. It is first found that the structural deformations in HfO_*x*_ nanofilm induced by migration of oxygen ions and interfacial electrochemical reactions can be recovered by OPT effectively. More importantly, such structural deformations no longer occur after OPT due to the strengthening in lattice structure, which directly illustrates the enhanced quantity of HfO_*x*_ nanofilm and the nature of enhanced RS properties after OPT. Finally, the underlying mechanisms of OPT-enhanced RS performance are analyzed by the results of X-ray photoelectron spectroscopic (XPS) surface analysis. In the OPT-enhanced HfO_*x*_ nanofilm, oxygen vacancies in crystalline regions can be remarkably reduced by active oxygen ions’ implantation. The oxygen ions transport will depend considerably on the grain boundaries and OPT-enhanced lattice structure will further guarantee the stability of conductive filaments, both of which ensure the uniformity and repeatability in RS processes. This study could provide a scientific basis for improving RS performance of oxides-based memristive devices by utilizing OPT.

## 1. Introduction

With the emergence of big data, cloud computing, the internet of things and artificial intelligence technology, the amount of information that needs to be stored and analyzed is exploding. However, as the mainstream in current semiconductor memory market, flash memory has encountered serious challenges to further scaling down [1]. Hence both industry and academia have exerted great efforts to explore appropriate candidates for the next-generation non-volatile memory (NVM) [2,3]. Due to the simple device structure, low operation voltages, fast operation speed, excellent scaling potential, multilevel data storage and uncomplicated fabrication process which is compatible with complementary metal-oxide-semiconductor (CMOS) technology, resistive random access memory (RRAM) with a simple metal-insulator-metal (MIM) structure is considered to be one of the most promising candidates for the next generation NVM [4,5]. RRAM can store information in the form of resistance states which rely on the history of applied voltage and can be reversibly switched. The resistive switching (RS) modes in RRAM devices can either be unipolar and bipolar. During bipolar RS mode, the devices transform from high-resistance state (HRS) to low-resistance state (LRS) at one polarity (defined as SET) and regain the original resistive state at reverse polarity (defined as RESET), whereas for the unipolar RS mode a change in polarity is not required [6]. A filamentary switching mechanism has been proposed to explain the RS phenomenon by the formation/rupture of nanoscale conductive filament (CF) [7]. As research continues, several well-accepted filamentary switching mechanisms have been raised to understand the underlying principle of the RS behaviors. They are respectively electrochemical metallization mechanism (ECM), thermochemical mechanism (TCM), valence change mechanism (VCM) and threshold switching (TS) for selector devices [1]. Moreover, the interfacial coupling mechanism (ICM) was also introduced to explain the RS between HRS and LRS [8].

Binary metal oxides such as TiO_2_, WO_3_, VO_2_, ZnO, Ta_2_O_5_, HfO_2_ and Fe_2_O_3_ have been extensively studied as RS layers due to their controllable components and simple structures [9,10,11,12,13,14,15]. Among various binary metal oxides, hafnium oxide (HfO_*x*_) shows distinguished advantages of large dielectric constant (~25), wide band-gap (~6 eV) and good thermal stability, leading to a potentially ultrathin storage layer with both enhanced function density and optical transparency [16]. Hence, HfO_*x*_-based memristive devices are promising candidates for future applications such as non-volatile memories, logic operations as well as artificial synapses in neuronal systems [16,17,18]. However, an uncontrolled diameter and number of CFs will cause poor uniformity and reliability of memristive devices. To address this problem, various approaches have been proposed to optimize the RS performance by modulating the growth and rupture of CFs, including device structure design [19,20], operating schemes optimization [21], and materials modulation [22,23].

In previous reports, defects and impurities in RS layers are the main factors that induce RS in RRAM devices. Therefore, the distribution of defects and impurities in RS layers can be controlled intentionally to improve the switching properties. Several methods have been investigated to modulate the RS layers’ materials, among which plasma treatment is proven to be a simple and effective way as well as impurity doping and crystallization modulating [24,25].

T. Kawashima et al. found that the RS performance in the SiO_2_/Cu stack could be modified by Ar plasma treatment in terms of operating voltages and light response, which were ascribed to oxygen vacancies produced in the surface region of the SiO_2_ by Ar plasma treatment [26]. Boncheol Ku et al. achieved the properties of forming-free process, faster switching speed, tighter low-resistance state and high-resistance state current distribution, smaller variations of SET/RESET voltages, and enhanced retention/endurance characteristics under HRS in HfO_2_-based RRAM through appropriate Ar plasma treatment [27]. For oxides-based memristive devices, the RS is presumably caused by the migration of oxygen ions, the concentration and distribution of oxygen ions/vacancies will affect the RS performance directly. Therefore oxygen plasma treatment (OPT) is considered to be a more effective method to modulate RS properties. For instance, Umesh Chand et al. suppressed the endurance degradation of HfO_2_-based RS memory by utilizing OPT which could increase extra available oxygen ions [28]. Xiaorong Chen et al. achieved improved RRAM stability due to the large quantity Ta_2_O_5_ near the top electrode after OPT, which played an important role in resistive switching of the devices [29]. Nevertheless, the lack of experimental evidence observed directly from the microscopic level of materials and appropriate understandings on how OPT modulates switching properties hinders its practical application.

In this paper, we revealed the OPT-enhanced RS performance in HfO_*x*_-based memristive devices by advanced conductive atomic force microscopy (C-AFM) combined with X-ray photoelectron spectroscopic (XPS) surface analysis. It was found that the structural deformations of the HfO_*x*_ nanofilm induced by migration of oxygen ions and interfacial electrochemical reactions can be eliminated by OPT effectively. Moreover, this type of deformations in HfO_*x*_ nanofilm no longer occur after OPT, which directly illustrates the enhanced quantity of HfO_*x*_ nanofilm and the nature of enhanced RS properties after OPT. Finally, we revealed the underlying mechanisms of the enhanced RS performance after OPT according to material analysis and electrical measurements.

## 2. Materials and Methods

### 2.1. Devices’ Fabrication

Starting from commercial SiO_2_/Ti/Pt (the thicknesses of Ti and Pt are 10 and 150 nm, respectively) wafers (1 cm × 1 cm), HfO_*x*_ nanofilm with a thick of 5 nm was deposited by radio frequency (RF) magnetron sputtering technique and utilizing high purity HfO_2_ ceramic (99.995%) as the target. During the magnetron sputtering process, the base pressure, RF power, direct-current (DC) bias and substrate temperature were 1 Pa, 60 W, 0 V and room temperature, respectively. High purity Ar (99.999%) and O_2_ (99.999%) with the O_2_/Ar ratio of 4:1 were introduced into the sample chamber during the HfO_*x*_ deposition process. The 50 nm-thick Pt top electrodes were directly deposited onto the HfO_*x*_ nanofilm through electron beam evaporation and patterned through a metal shadow mask. A pressure of ~10^−6^ Pa and a deposition rate of ~0.5 Å/s at room temperature were controlled during the Pt deposition process. Oxygen plasma treatment was performed by an inductively coupled plasma (ICP) reactor which supplied a RF power of 400 W to the upper electrode and 100 W to the lower electrode. During the plasma exposure, the gas flow rate and oxygen pressure were 50 sccm and 2 Pa, respectively. The plasma exposure time was 30 s.

### 2.2. Characterization

The C-AFM measurements of the HfO_*x*_ nanofilm were performed by using an AFM (Cypher S, Oxford Instruments Inc., Santa Barbara, CA, USA). The sample was probed by a Ti/Ir tip (ASYELEC-01) in contact mode to carry out C-AFM measurements, which the DC voltage was applied onto the tip. The topographic images were achieved in contact mode with no voltage applied to the tip. All electrical properties of the HfO_*x*_ based memristive devices were characterized at room temperature and in an atmospheric environment. The current-voltage (*I*-*V*) characteristics of the Pt/HfO_*x*_/Pt structures were measured on a semiconductor parameter analyzer (Agilent B1500A, Agilent Technologies Inc., Santa Clara, CA, USA). During the *I-V* measurements, Pt bottom electrodes were always grounded and the bias voltages were applied to the top electrode. All transmission electron microcscope (TEM) specimens were fabricated by a dual beam focus ion beam (FIB, FEI Helios 450S, Nanolab Technologies Inc., Milpitas, CA, USA) workstation with the dimension of 500 nm height × 3 μm width × 40 nm thickness. The cross-sectional high-resolution transmission electron microscopic (HRTEM) images and scanning transmission electron microscope (STEM) with energy-disperse spectra (EDS) images were obtained on a TEM (FEI Titan Themis 200, Thermo Fisher Inc., Waltham, MA, USA) In order to investigate the film composition and oxidative states of the HfO_*x*_ before and after OPT, surface XPS (ESCALAB 250Xi, Thermo Fisher Inc., Waltham, MA, USA) analysis of the samples were performed. A monochromatic Al-Kα X-ray source (1486.6 eV photons) was used. A pass energy of 30 eV was employed for the core-level spectra scan. The X-ray source was run at a reduced power of 150 W. The pressure in analysis chamber was maintained at 10^−9^ mbar during measurements. The core-level signals were recorded at a photoelectron take-off angle (α, measured with respect to the sample surface) of 90°. All binding energies (BEs) were referenced to C 1s hydrocarbon peak at 284.6 eV. In curve fitting, the line width (full width at half-maximum, FWHM) for the Gaussian peaks was maintained constant for all components in particular spectrum. Surface elemental stoichiometries were determined from XPS spectral area ratios and were reliable within ± 5%. The elemental sensitivity factors were calibrated using stable binary compounds of well-established stoichiometries. Background correction and peak fit features were performed to the data using Avantage software.

## 3. Results and Discussion

As the development of emerging nanoionics, ions transport process and related electrochemical reactions at nanoscale have been studied extensively [30,31]. According to previous studies, ions can easily diffuse by vacancies and transport along grain boundaries [32,33]. As a result, the formation, rupture and reaggregation of CFs in polycrystalline HfO_*x*_ nanofilm-based memristive devices may be stochastic, which will cause inhomogenous programming parameters. Therefore, efforts can be devoted to modulating the electronic structure of polycrystalline HfO_*x*_ nanofilm by carefully adjusting the concentration and distribution of oxygen ions/vacancies.

**Figure 1:**
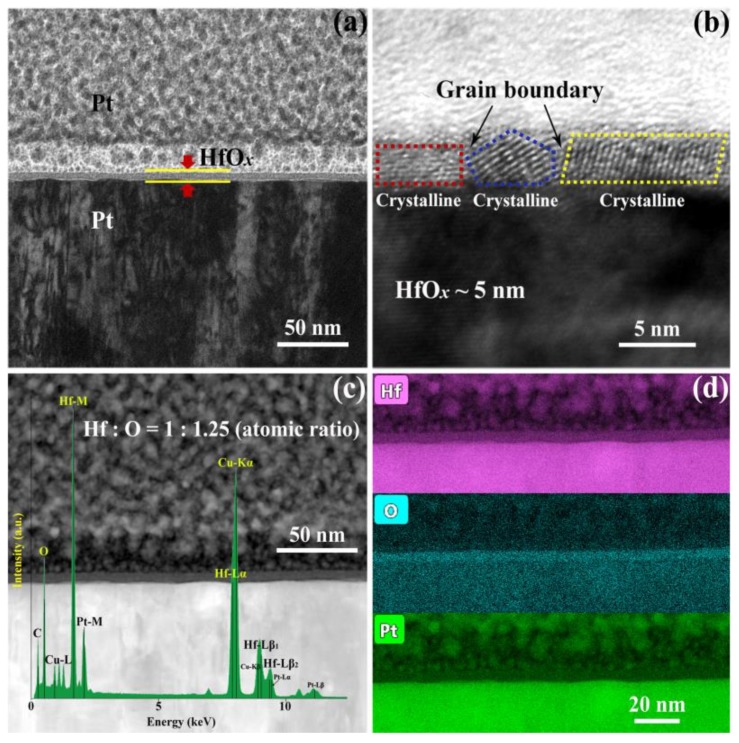
Cross-sectional (**a**) transmission electron microscope (TEM) image and (**b**) high-resolution transmission electron microscopy (HRTEM) image of the Pt/HfO_*x*_/Pt resistive switching (RS) device. (**c**) High-angle annular dark-field scanning transmission electron microscopy (HAADF STEM) image with energy-disperse spectra (EDS) analysis in the HfO_*x*_ RS layer and (**d**) EDS mappings of the Hf, O and Pt distribution edges.

In order to investigate the influence of OPT on prepared HfO_*x*_ nanofilm, a series of electrical measurements and material analysis were carried out. The HfO_*x*_ nanofilm with the thickness of 5 nm was firstly deposited onto a commercial Pt/Ti/SiO_2_ substrate by the RF magnetron sputtering technique, as visualized by the cross-sectional TEM image in Figure 1a. Then the as-prepared HfO_*x*_ nanofilm was observed by HRTEM as shown in Figure 1b, we can clearly see that the HfO_*x*_ nanofilm exhibits a polycrystalline structure with typical grain boundaries (Figure S1 shows the topographic AFM image of grains in the as-deposited HfO_*x*_ nanofilm). The electrical transport characteristics of the RS layer can be affected by its element compositions significantly. Therefore, the high-angle annular dark-field scanning transmission electron microscopy (HAADF STEM) image and energy-dispersive spectroscopy (EDS) mappings of the HfO_*x*_ nanofilm are presented in Figure 1c,d, respectively. One can see that the atomic ratio of hafnium and oxygen in HfO_*x*_ nanofilm is nearly 1:1.25, indicating that the as-grown HfO_*x*_ nanofilm possesses vast oxygen vacancies which will cause unstable lattice structure and uncontrollable CFs during the RS processes.

**Figure 2:**
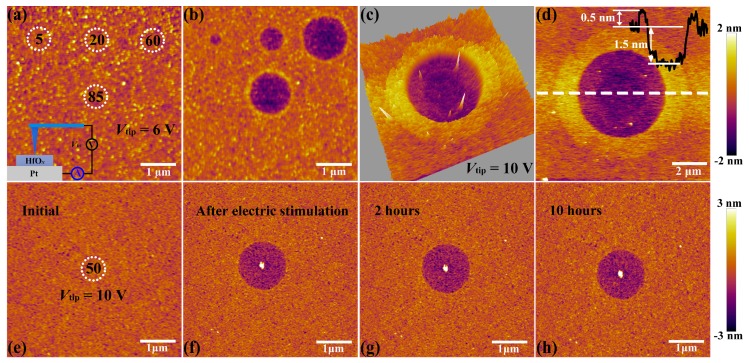
(**a**) Topographic atomic force microscopy (AFM) image of the locations (white dotted circle) where voltage sweepings were performed with the amplitude of 6 V for 5, 20, 60, 85 times, respectively (insert shows the schematic of the conductive atomic force microscopy (C-AFM) measurements). (**b**) Topographic AFM image of the HfO_*x*_ nanofilm after stimulated by voltage sweepings up to 6 V during preceding C-AFM measurements for 5, 20, 60, 85 times, respectively. (**c**) Three dimensional and (**d**) two dimensional topographic images of the HfO_*x*_ nanofilm after stimulated by C-AFM with a voltage sweepings of 10 V in an area of 1 × 1 μm^2^. Insert shows the height and depth of HfO_*x*_ nanofilm from the AFM cross-sectional profile along the white dotted line. (**e**) Topographic image showing the position where voltage sweepings were performed with the amplitude of 10 V for 50 times. (**f**–**h**) Topographic images after removing the electrical stimuli for 0, 2, 10 h, respectively.

The C-AFM measurements were performed by utilizing a Ti/Ir coated conductive tip in contact mode as a top microelectrode, as shown in the insert part of Figure 2a. Then DC voltage sweepings from 0 to 6 V with a compliance current (*I*_cc_) of 20 nA were subjected onto the tip at four positions for 5, 20, 60 and 85 times, respectively (see Figure 2a). It is obvious that structural deformations took place at the positions of applied electric stimulation (see Figure 2b). To be more specific, the diameter and depth of circular pits increase from 0.3 to 1.3 μm and 0.7 to 1.5 nm respectively, corresponding to the sweeping cycles increase from 5 to 60 times. It is worth noting that the shape and dimension of circular pits cannot enlarge continuously when it reaches a certain limit (comparing the sweeping cycles between 60 and 85 times), which is attributed to the constraint of local electric field, indicating that this process is driven by the applied electric field. Such structural deformations have been studied to be a result of oxygen ions migration and subsequent oxygen gas eruption induced by electrochemical reactions at the top interface between conductive tip and HfO_*x*_ nanofilm, which also result in the accumulation of vast interstitial oxygen atoms and oxygen vacancies near these circular pits [34,35]. To further explore the localization of oxygen ions’ motion and related electrochemical reactions, we applied a DC voltage bias of 10 V onto the conductive tip to scan a region of 1.0 × 1.0 μm^2^ for 5 times with the scan rate of 1 Hz in contact mode. Soon afterwards, an extended area of 10 × 10 μm^2^ was scanned with no bias applied to the tip. As shown in Figure 2c,d, more drastic oxygen ions’ migration, accumulation and related electrochemical reactions at the top interface were detected, which result in the severer collapse of lattice structure with the diameter of circular pit up to about 5 μm. This phenomenon illustrates that the oxygen ions’ migration and related electrochemical reactions are indeed induced by the external electric field rather than the particularity of selected regions. The prominent ring around the middle pit may be attributed to the lattice distortion that resulted from interstitial oxygen atoms and vacancies which can induce expansion of lattice structure around the central region under the external electric field.

**Figure 3:**
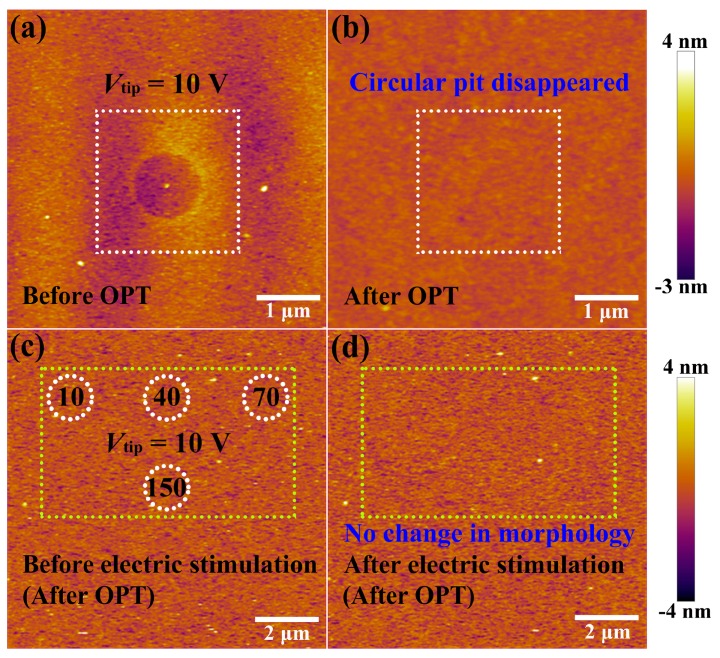
(**a**) Topographic image of the as-deposited HfO_*x*_ nanofilm with a circular pit created by electrical stimuli before oxygen plasma treatment (OPT) and (**b**) the circular pit disappeared after OPT (see the the white dashed box). After OPT, (**c**) topographic image before applying electrical stimuli (white dotted circles indicate the locations where voltage sweepings will be performed) and (**d**) no morphological changes after applying electrical stimuli (see the the yellow dashed box).

Next, the HfO_*x*_ nanofilm with a circular pit which is created by the migration of oxygen ions accompanied with occurrence of electrochemical reactions and generation of oxygen gas in the external electric field was treated by oxygen plasma with exposure time of 30 s. It is found that structural deformations of the HfO_*x*_ nanofilm induced by electrical stimuli were repaired by OPT (see Figure 3b), compared with the morphology before OPT in Figure 3a (the positioning method before and after OPT was presented in Figure S2). To illustrate the disappearance of circular pit is the effect of OPT instead of selfreparing without any external field, we investigated the retentivity of the circular pit. As depicted in Figure 2e, C-AFM measurements were performed on the sample with DC voltage sweepings to 10 V at the central location for 50 times. Figure 2f–h show topographic measurements 0, 2 and 10 h after removing the electrical stimulation, respectively. One can see that the structural deformations occurred on the sample still exist even after 10 h. It is necessary to declare that the total time of the OPT process is less than 2 h, including placing the sample, pumping vacuum, setting up parameters, etc. Hence we can confirm that the repairation of structural deformations is attributed to OPT. The reparation of a circular pit can be understood as a chemical reaction, which is the recombination of oxygen ions and oxygen vacancies or the formation of Hf–O bonds. To further illuminate the physical nature of the repairation phenomenon in HfO_*x*_ nanofilm after OPT, the same C-AFM measurement procedures were applied to the oxygen plasma-treated HfO_*x*_ nanofilm. DC voltage sweepings from 0 to 10 V with a *I*_cc_ of 20 nA were applied to the tip at four positions for 10, 40, 70 and 150 times, respectively (see Figure 3c). Remarkably, we can see that there is no structural deformations took place at the positions where electric stimulation was applied (see Figure 3d). On the basis of previous discussions, oxygen vacancies in polycrystal HfO_*x*_ nanofilm could be partly filled with oxygen ions after OPT, which resulted in the more stable lattice structure. More specifically, oxygen vacancies near the top surface of the HfO_*x*_ nanofilm are easier to fill than the deeper ones due to the energy loss of oxygen ions in oxygen plasma with the increasing implantation depth, which ensures the more robust lattice structure at the top surface than the deeper regions. Therefore, the physical deformations that occurred at the mechanically weakest part of the thin films cannot arise in the HfO_*x*_ nanofilm after OPT due to the more stable lattice structure especially near the top interface [35].

**Figure 4:**
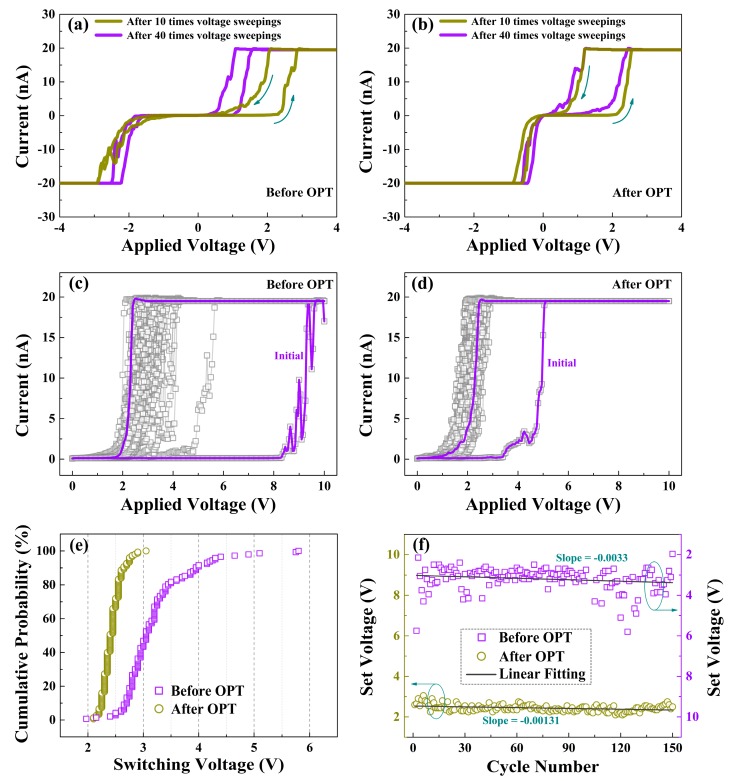
Typical *I*-*V* curves of the as-deposited HfO_*x*_ nanofilm measured by in situ C-AFM at *I*_cc_ of 20 nA after 10, 40 times voltage sweepings respectively, (**a**) before OPT showing an excursion in *I-V* curves and (**b**) after OPT showing no excursion in *I-V* curves. SET processes curves of (**c**) the as-deposited HfO_*x*_ nanofilm and (**d**) the HfO_*x*_ nanofilm after OPT. (**e**) Cumulative probability of *V*_SET_ in HfO_*x*_ nanofilm before and after OPT. (**f**) Linear fitting of *V*_SET_ in HfO_*x*_ nanofilm before and after OPT from 150 switching times.

To further illustrate the influence of OPT on the switching characteristics of HfO_*x*_ nanofilm, the C-AFM measurements were performed with a DC voltage was applied to the Ti/Ir-coated Si tip and the bottom Pt electrode was grounded. Through in situ morphology and electrical measurements, we primarily researched the influence of structural deformations mentioned above on the electrical properties of the as-prepared HfO_*x*_ nanofilm. With the pretreatment of applying 10 V voltage sweepings for 10, 40 times respectively, we subsequently applied the voltages (0 to 6 V, 6 to 0 V, 0 to −6 V, −6 to 0 V) onto the tip to measure the *I-V* curves under the two pretreatment conditions, respectively. Based on the discussions above, the pretreatment of 40 times produced larger structural deformations than 10 times, suggesting more oxygen vacancies generated by the pretreatment, which enhanced the conductance of HfO_*x*_ nanofilm. Therefore, the lower switching voltages were obtained for the pretreatment of 40 times than 10 times (see Figure 4a). However, we observed no remarkable excursion in *I-V* curves when the same operations were applied on the HfO_*x*_ nanofilm after OPT (see Figure 4b), which can be accounted for by the more stable lattice structure. As plotted in Figure 4c, the SET curves of the as-grown HfO_*x*_ nanofilm show a significant fluctuation, which mainly caused by the continuous generation of oxygen vacancies at the area of circular pit induced by electrochemical reactions, which changes the conductance of CFs in HfO_*x*_ nanofilm after each SET process. Nevertheless, after OPT, as shown in Figure 4d, the HfO_*x*_ nanofilm exhibits a more stable SET process. The lower *V*_SET_ can be explained by the migration of non-lattice oxygen ions supplied by OPT, which facilitates the arrival of *I*_cc_. Figure 4e presents the *V*_SE__T_ cumulative probability of the HfO_*x*_ nanofilm before and after OPT. We can observe clearly that the distribution of statistical *V*_SET_ after OPT is more concentrated than that before OPT. In addition, Figure 4f gives the linear fitting of extracted *V*_SET_ before and after OPT from SET sweepings for 150 times, the decrease of |slope| of *V*_SET_ from 0.0033 to 0.00131 after OPT implies not only the generation of oxygen vacancies near the circular pit before OPT but also the positive effect of an undamaged structure during the electric stimulation after OPT. The significant improvement of uniformity in SET processes after OPT can be attributed to the more stable lattice structure, which avoided physical disruption during the SET processes [35].

**Figure 5:**
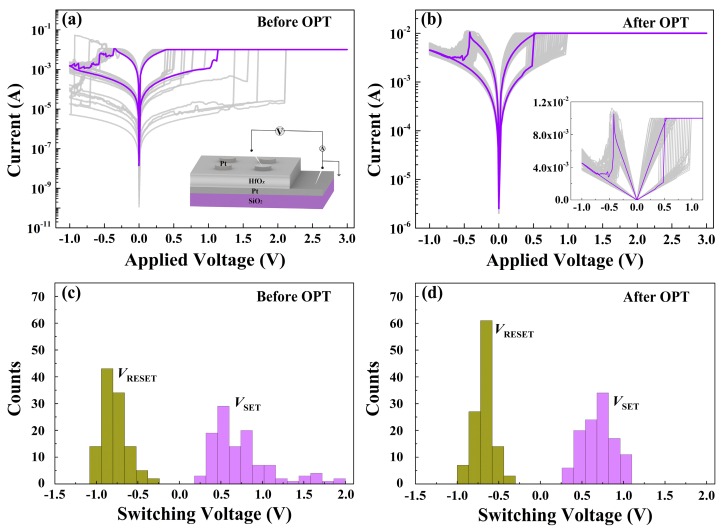
(**a**) RS characteristics of the Pt/HfO_*x*_/Pt device before OPT (insert shows the schematic of the memory device). (**b**) RS characteristics of the Pt/HfO_*x*_/Pt device after OPT. Histograms of the SET/RESET switching voltages in the Pt/HfO_*x*_/Pt memory devices (**c**) before OPT and (**d**) after OPT from over 110 direct-current (DC) RS cycles.

The enhanced RS characteristics were also found in the prepared HfO_*x*_-based memristive devices after OPT. As plotted in Figure 5a, the Pt/HfO_*x*_/Pt device before OPT shows typical bipolar RS behaviors in *I*-*V* curves, displaying a dramatic fluctuation (insert part shows the schematic of the memory device). Oxygen gas bubbles which were caused by oxygen ions’ migration and related electrochemical reactions were observed in the top Pt electrode of Pt/as-deposited HfO_*x*_/Pt device during RS processes (see Figure S3) [35]. After OPT, we found that the RS curves show a much better stability and repeatability (see Figure 5b). The histograms and statistical charts in Figure 5c,d show the switching voltage distributions of the Pt/HfO_*x*_/Pt devices without/with OPT obtained from the RS curves, respectively. The switching voltages of the devices without OPT are distributed in widespread ranges of 0.2 to 2 V (*V*_SET_) and −0.2 to −1 V (*V*_RESET_), while the switching voltages of the devices with OPT are distributed in scales of 0.3 to 1.1 V (*V*_SET_) and −0.3 to −0.5 V (*V*_RESET_). Obviously, the switching voltages of the Pt/HfO_*x*_/Pt devices with OPT are much more stable than those without OPT. Also, the decrease of switching voltages can be attributed to the participation of non-lattice oxygen ions supplied by OPT during the RS processes which are conductive to the forming of the fine conductive filaments.

**Figure 6:**
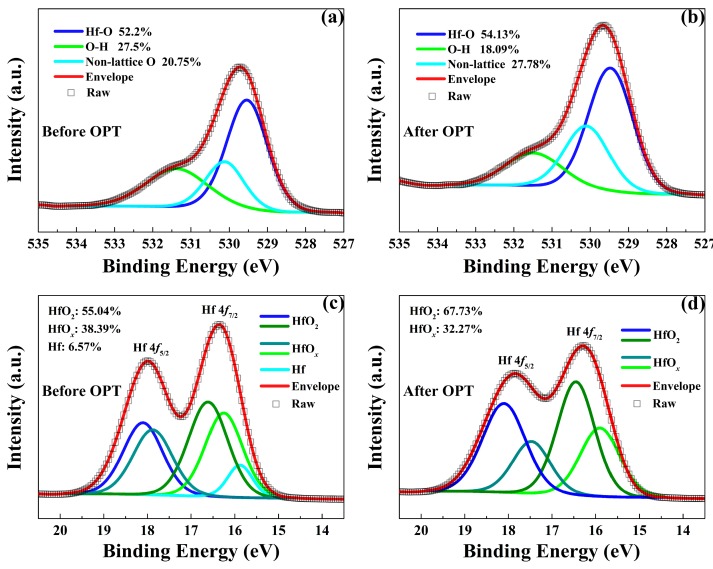
X-ray photoelectron spectroscopy (XPS) spectra of O 1s in the HfO_*x*_ nanofilm (**a**) before OPT and (**b**) after OPT. XPS spectra of Hf 4f in the HfO_*x*_ nanofilm (**c**) before OPT and (**d**) after OPT.

As shown in Figure 6, XPS measurements were performed to further explain the physical nature of the enhanced RS performance observed in the HfO_*x*_-based memristive devices after OPT. Figure 6a,b show the XPS spectra of O 1s in the as-grown HfO_*x*_ nanofilm before and after OPT, respectively. The O 1s signal with the binding energy of 529.5 eV are mainly arising from the lattice oxygen (Hf-O bonding, HfO_2_ components). While the higher binding energy of O 1s at 530.14 eV are associated with the non-lattice oxygen anions. One can see that lattice oxygen species increase from 52.2% to 54.31% and non-lattice oxygen also increases from 20.75% to 27.78%. In addition, Hf 4f XPS spectra also provide essential information of the composition change in HfO_*x*_ nanofilm after OPT. As shown in Figure 6c,d, the presence of the Hf 4f_7/2_ and Hf 4f_5/2_ species with the binding energies of 16.29 eV and 17.9 eV corresponds to the predominant HfO_*x*_ components where *x* is less than 2, while the minor high binding energy Hf 4f_7/2_ and Hf 4f_5/2_ species at 16.6 eV and 18.1 eV are associated with the HfO_2_ components. In addition, the binding energy Hf species at 16 eV correspond to metallic Hf. After OPT, the HfO_2_ species increase from 55.04% to 67.73%, the HfO_*x*_ species decrease from 38.39% to 32.27% where *x* is less than 2 and the metallic Hf disappeared. The above XPS results indicate that the OPT can increase the number of non-lattice oxygen and the concentration of HfO_2_ which improved the stability of lattice structure.

**Figure 7:**
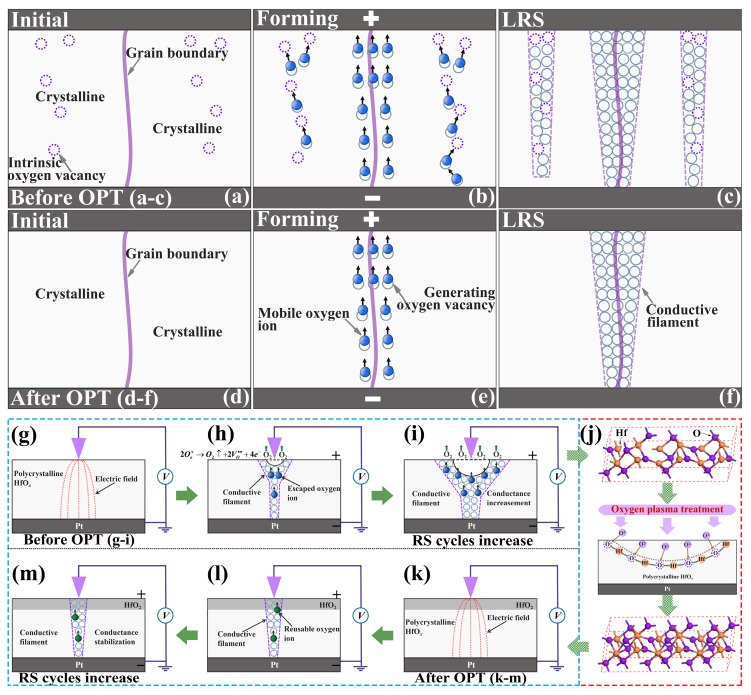
Schematic diagrams for the mechanism of oxygen ions migration in Pt/HfO_*x*_/Pt devices, (**a**–**c**) before OPT and (**d**–**f**) after OPT. Schematic diagrams for the variation of CFs induced by the structural deformations in the HfO_*x*_ nanofilm, (**g**–**i**) before OPT and (**k**–**m**) after OPT. Particularly, (**j**) gives a schematic physical process of OPT on the HfO_*x*_ nanofilm.

Considering all the above experimental results, the mechanisms of the RS properties’ improvement in the oxygen plasma-treated Pt/HfO_*x*_/Pt devices were proposed based on the theory of CFs. Figure 7 schematically show the processes of RS behaviors in a single device. The as-grown polycrystalline HfO_*x*_ possesses a large amount of intrinsic vacancies in crystalline regions (violet dotted circle in Figure 7a), which is consistent with aforementioned EDS and XPS analysis. According to previous reports, when a positive voltage is applied to the inert Pt top electrode while the Pt bottom electrode is grounded, oxygen ions will be attracted to the top interface, subsequently the CFs consisting of oxygen vacancies will be formed mainly along the grain boundaries in polycrystalline HfO_*x*_ nanofilms [32]. Nevertheless, a certain amount of oxygen vacancies apart from grain boundaries can also provide transport routes for oxygen ions in polycrystalline HfO_*x*_ nanofilm. Hence we can believe that multiple and stochastic CFs will be formed not only along grain boundaries but also the location beyond grain boundaries but rich in oxygen vacancies after electric stimuli. During the forming process, oxygen ions will not only transport along the grain boundaries but also by means of the oxygen vacancies (see Figure 7b). As a result, the stochastic forming of the multiple CFs will lead to severe fluctuation of the programming voltages and the device resistances (see Figure 7c). In the oxygen plasma-treated Pt/HfO_*x*_/Pt device, oxygen vacancies in crystalline regions drastically reduced by the active oxygen ions’ implantation (see Figure 7d), as proved by the XPS analysis and AFM experiments above. Therefore, the oxygen ions’ transport depends considerably on the grain boundaries in polycrystalline HfO_*x*_ nanofilm [36]. During the forming process, oxygen ions transport through the assistance of intrinsic oxygen vacancies in crystalline regions will be restrained effectively (see Figure 7e). As a result, the CFs will be formed mainly along the grain boundaries (see Figure 7f), and better stability and uniformity will be achieved in RS processes. During the RESET process, the increased non-lattice oxygen can migrate back to recombine with oxygen vacancies which increase the probability of the recombination, promoting the stability of the RESET process [28]. It is worth noting that the transport of non-lattice oxygen during the RS processes also relies considerably on the grain boundaries, hence, this type of transport can not cause additional instability. 

Last but not least, the significant fluctuation in the Pt/as-deposited HfO_*x*_/Pt device also partly due to the continuous generation of oxygen vacancies at the area of the circular pit which is the mechanically weakest part of the HfO_*x*_ nanofilm forming CFs during RS cycles. The continuous generation of oxygen vacancies increase the conductance of CFs as the increasing sweeping cycles (see Figure 7g–i), which is consistent with the former C-AFM topographic measurements (see Figure 2b). Through OPT (see Figure 7j), the increase of HfO_2_ components especially near the top interface ensures a more stable lattice structure and eliminates the physical structural deformations effectively, which were proved by direct experimental evidence from microscopic level of materials (see Figure 3b,d). Therefore, the conductance of CFs can remain stable during the RS processes (see Figure 7k–m), which further ensures the uniformity and repeatability in RS processes. Overall, after OPT, the more stable lattice structure and increased available non-lattice oxygen improve the RS properties of the HfO_*x*_-based memristive devices.

## 4. Conclusions

In summary, we directly observed the recovery phenomenon of structural deformations induced by migration of oxygen ions and interfacial electrochemical reactions in HfO_*x*_ nnaofilm after OPT through in situ scanning probe microscope (SPM) technologies. In addition, such structural deformations no longer occur due to the strengthening in lattice structure, which demonstrates the enhanced quantity of HfO_*x*_ nanofilm after OPT. These results provide direct experimental evidence from a microscopic level of materials for OPT-enhanced RS performance. Furthermore, we proposed possible mechanisms for the enhanced RS properties in Pt/HfO_*x*_/Pt devices after OPT. On the one hand, the OPT-enhanced lattice structure with no structural deformations under the electric stimulation can guarantee the stability of CFs. On the other hand, in the OPT-enhanced HfO_*x*_ nanofilm, oxygen vacancies in crystalline regions can be remarkably reduced by the active oxygen ions’ implantation, and the oxygen ions will transport depends considerably on the grain boundaries. Besides, a mass of OPT-supplied non-lattice oxygen ions can not only boost a fine size of conductive filaments but also take part in the RESET processes continuously. As a result, the uniformity and repeatability of RS characteristics are improved. We expect these findings could help in the understanding of the physical nature of OPT-enhanced RS properties and provide a scientific basis for improving the switching performance of oxides-based memristive devices by utilizing OPT.

## Supplementary Materials

The following are available online at https://www.mdpi.com/2079-4991/9/10/1355/s1, Figure S1: Topographic AFM image of grains in the as-deposited HfO_*x*_ nanofilm, Figure S2: Topographic AFM images of the as-deposited HfO_*x*_ nanofilm for positioning, (a) initial, (b) after electrical stimuli and (c) after OPT, Figure S3: Optical microscope images of the Pt top electrode in Pt/as-deposited HfO_*x*_/Pt device (a) before and (b) after resistive switching (RS) measurements.
