# Supplementary material for: Direct Observation of Structural Deformation Immunity for Understanding Oxygen Plasma Treatment-Enhanced Resistive Switching in HfOx-Based Memristive Devices
Source: Nanomaterials (Basel). 2019 Sep 21;9(10):1355. doi: 10.3390/nano9101355 (PMC6836033; doi:10.3390/nano9101355)
Supplement: Supplementary file 1 [file nanomaterials-09-01355-s001.pdf]

Direct Observation of Structural Deformation Immunity for Understanding Oxygen Plasma Treatment-Enhanced Resistive Switching in HfO_x-Based Memristive Devices

Dong Wang [1], Shaoan Yan [1,2,*], Qilai Chen [1], Qiming He [3], Yongguang Xiao [4], Minghua Tang [4] and Xuejun Zheng [1,2,*]

[1] School of Mechanical Engineering, Xiangtan University, Xiangtan, Hunan 411105, China
[2] Key Laboratory of Welding Robot and Application Technology of Hunan Province, School of Mechanical Engineering, Xiangtan University, Xiangtan, Hunan 411105, China
[3] Key Laboratory of Microelectronics Devices and Integration Technology, Institute of Microelectronics, Chinese Academy of Sciences, Beijing 100029, China
[4] School of Materials Science and Engineering, Xiangtan University, Xiangtan, Hunan, 411105, China
* Correspondence: yanshaoan@xtu.edu.cn (S.Y.), zhengxuejun@xtu.edu.cn (X.Z.)

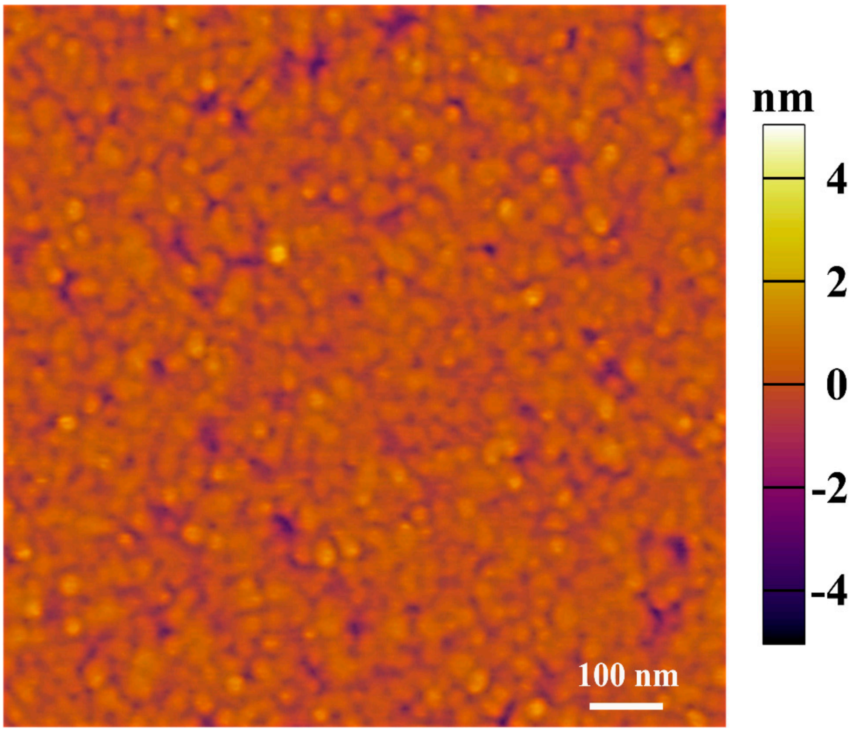

Figure 1. Topographic AFM image of grains in the as-deposited HfO_x nanofilm.

Figure S1 gives the topographic AFM image of grains in the as-deposited HfO_x nanofilm, which reveals a granular corrugation with an RMS roughness of 0.38 nm over an area of 1 μm^2. Such a flat film surface and uniform grain size distribution ensure the validity and accuracy of the CAFM measurement. Conductive paths are usually associated with grain boundaries in which the abundant presence of defects and oxygen vacancies leads to the formation of localized states and bands within the HfO_x bandgap.[1]

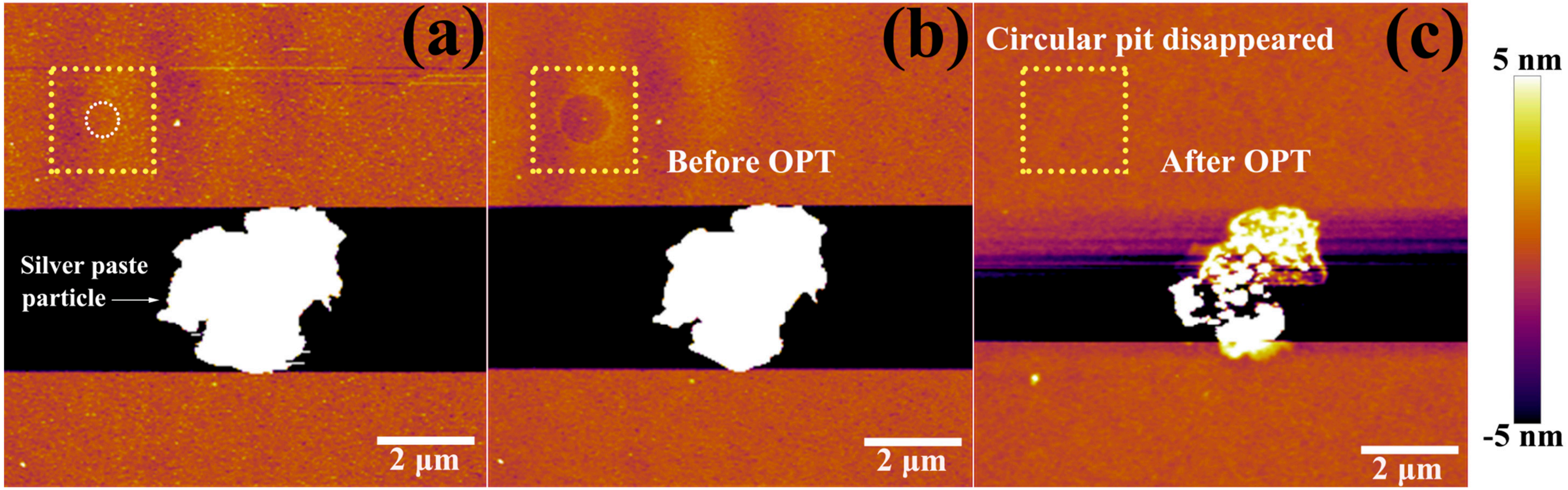

Figure 2. Topographic AFM images of the as-deposited HfO_x nanofilm for positioning, (**a**) initial, (**b**) after electrical stimuli and (**c**) after OPT. .

Figure S2 shows the positioning method of AFM topography scanning in the same area before and after OPT. Here, the silver paste microparticle was dropped on the surface of the HfO_x nanofilm by using a capillary tube.[2] We can see that the morphology of the silver particle before and after OPT is very easy to distinguish, this positioning method is more convenient, no mask and metal evaporation are required, and the effect of heat treatment on OPT is avoided.

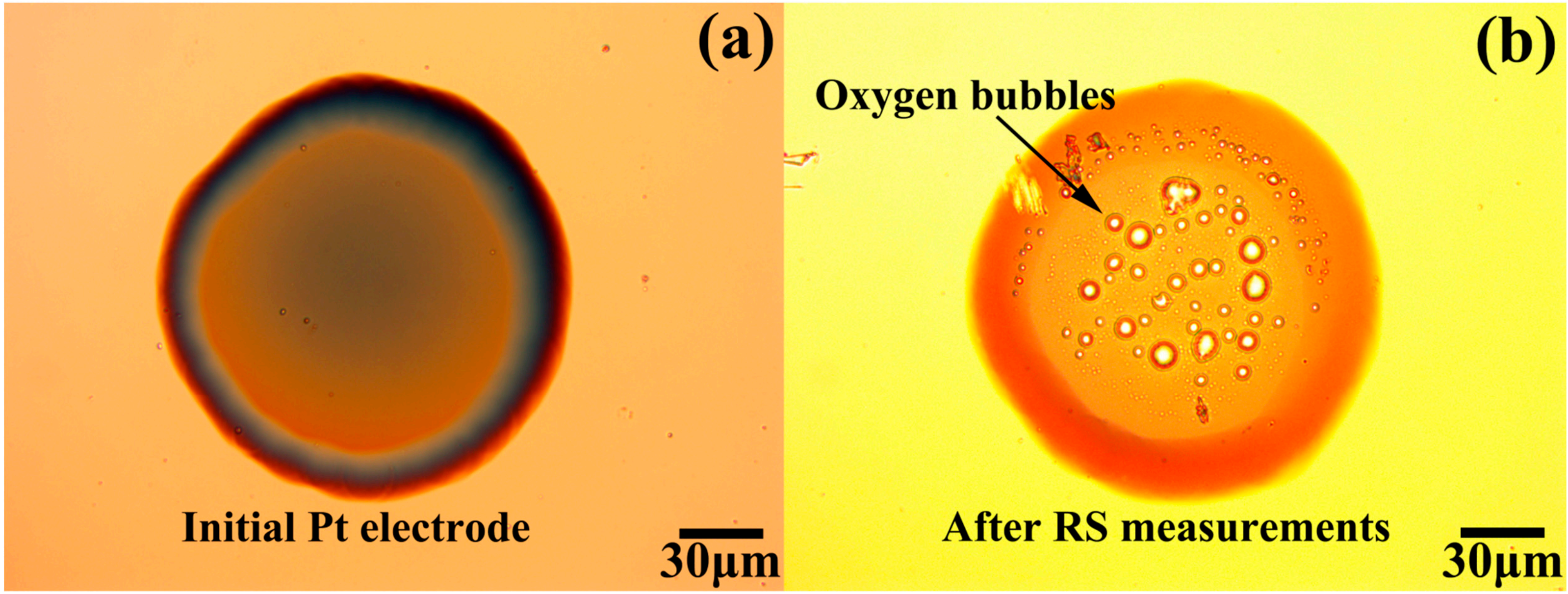

Figure 3. Optical microscope images of the Pt top electrode in Pt/as-deposited HfO_x/Pt device (**a**) before and (**b**) after resistive switching (RS) measurements.

As shown in **Figure S3**, we can clearly see that a lot of oxygen bubbles are generated on the Pt electrode after the RS measurements, which is due to the migration of oxygen ions driven by the electric field and the continuous electrochemical reactions at the surface. The creation of oxygen gas is accompanied by the concomitant creation of oxygen vacancies in the HfO_x nanofilm, indicating the occurrence of the structural deformations and damages.[3]

References

1 S. Brivio, G. Tallarida, E. Cianci and S. Spiga, *Nanotechnology*, 2014, **25**, 385705 (7pp).

2 L. Hirt, A. Reiser, R. Spolenak and T. Zambelli, *Adv. Mater.*, 2017, **29**, 1604211.

3 J. J. Yang, F. Miao, M. D. Pickett, D. A. A. Ohlberg, D. R. Stewart, C. N. Lau and R. S. Williams, *Nanotechnology*, 2009, **20**, 215201.
